# From one to many: expanding the *Saccharomyces cerevisiae* reference genome panel

**DOI:** 10.1093/database/baw020

**Published:** 2016-03-17

**Authors:** Stacia R. Engel, Shuai Weng, Gail Binkley, Kelley Paskov, Giltae Song, J. Michael Cherry

**Affiliations:** Department of Genetics, Stanford University, Stanford, CA, 94305

## Abstract

In recent years, thousands of *Saccharomyces cerevisiae* genomes have been sequenced to varying degrees of completion. The *Saccharomyces* Genome Database (SGD) has long been the keeper of the original eukaryotic reference genome sequence, which was derived primarily from *S. cerevisiae* strain S288C. Because new technologies are pushing *S. cerevisiae* annotation past the limits of any system based exclusively on a single reference sequence, SGD is actively working to expand the original *S. cerevisiae* systematic reference sequence from a single genome to a multi-genome reference panel. We first commissioned the sequencing of additional genomes and their automated analysis using the AGAPE pipeline. Here we describe our curation strategy to produce manually reviewed high-quality genome annotations in order to elevate 11 of these additional genomes to Reference status.

**Database URL**: http://www.yeastgenome.org/

## Introduction

Recent advances in sequence technology have led to an explosion of available sequence data, and thousands of *Saccharomyces cerevisiae* genomes have been sequenced to varying degrees of completion in just the past few years (1). These genomes come from a variety of laboratory and commercial strains, as well as from clinical and environmental isolates. More than 100 of these genomes have been assembled to the level of scaffold or chromosome (see the Genome database at the National Center for Biotechnology Information (NCBI); http://www.ncbi.nlm.nih.gov/genome/genomes/15), and have been deposited in the primary sequence databases (GenBank, ENA, DDBJ) that make up the International Nucleotide Sequence Database Collaboration (INSDC; http://www.insdc.org).

The *Saccharomyces* Genome Database (SGD; http://www.yeastgenome.org/) has long been the keeper of the first eukaryotic genome sequence, which was derived primarily from *S. cerevisiae* strain S288C and published in 1996 as the output of several years of collaborative work of an international consortium of researchers (2). This ‘reference genome’ was produced to serve as a single consensus representative *S. cerevisiae* genome sequence against which all other sequences could be measured. Since that time, SGD has made that original eukaryotic reference sequence and its annotation freely available to researchers around the world, who have thoroughly studied the sequence and put it to use in many great scientific discoveries and breakthroughs (3).

We are now firmly in the modern era of yeast genomics, in which the rich variety of available genomic sequences has changed the way we study genomes. Comparative genomics is providing clear pictures of the full constituent parts of species’ genomes, which vary not only in nucleotide sequence, but also in gene complements and chromosome architecture. Although much work in yeast genomics has focused on S288C (4) and its derivatives (5), a number of different strains are more informative for distinct areas of study [e.g. W303 for aging (6), SK1 for meiosis and sporulation (7)], and are popularly used in research because their distinct phenotypes.

**Figure 1.**
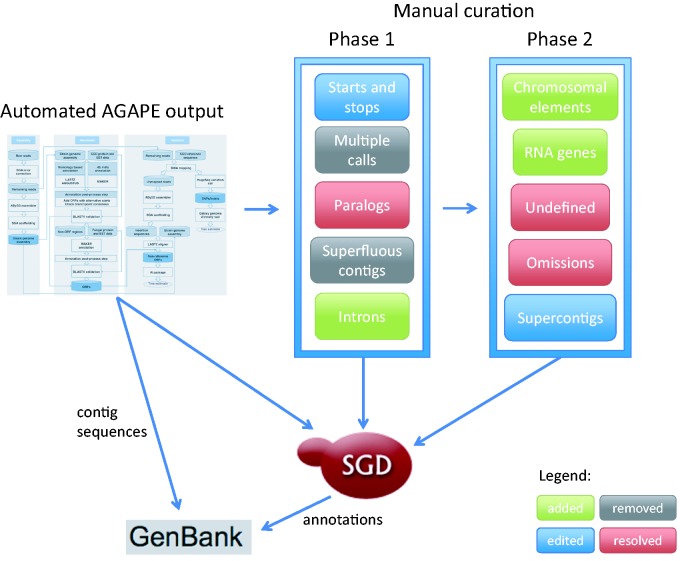
Curation strategy currently in use at SGD to expand the original *Saccharomyces cerevisiae* systematic reference sequence from a single highly-curated genome to an expertly curated multi-genome reference panel.

New technologies have been quickly pushing *S. cerevisiae* annotation well past the limits of any system based exclusively on a single reference sequence. Therefore, SGD is actively expanding its sequence curation efforts to provide a 12-genome reference panel that allows us to more comprehensively annotate the genetic background studied in experiments for which a strain of known provenance is reported. The 11 additional high-profile yeast genomes were selected because they are widely studied, and have sizable amounts of published experimental and phenotypic data available. When combined with high quality sequence information, these detailed phenotype and functional annotations will allow researchers to fully understand the specific genomic basis of phenotypic variation. Here we present a description of the curation strategy currently in use to expand the original *S. cerevisiae* systematic reference sequence from a single highly-studied genome to an expertly curated multi-genome reference panel, as shown in Figure 1.

## Materials and Methods

We recently updated the S288C reference sequence (8), and then commissioned the sequencing of additional genomes and their automated analysis using the Automated Genome Analysis PipelinE (AGAPE) pipeline (9; NCBI BioProject Accession PRJNA260311). AGAPE accepts raw sequence reads as input, generates *de novo* assembly scaffolds and contigs, then integrates the steps of open reading frame (ORF) annotation and sequence variation calling. The original AGAPE output is available at http://downloads.yeastgenome.org/published_datasets/Song_2015_PMID_25781462/. We are now applying the following curation strategy to produce manually reviewed high-quality genome annotations in order to elevate 11 of these additional genomes to Reference status. The 11 strains from which these genome sequences were derived have recently been described elsewhere (1, 9). These strains were selected because they have a substantial history of use and experimental results, and also because they are the genomes for which we have the most curated phenotype data and for which we aim to curate specific functional information.

### The curation strategy

Step 1: boundary differences. We first identified ORF calls for which the predicted translation start and/or stop differed from the S288C reference, then reviewed them manually and corrected as necessary. These annotation changes and ORF revisions were performed in the same manner that we have used in the past to update the S288C systematic reference sequence, and which we have described previously (10). For the 11 strains, a total of 1009 such differences were identified (Table 1). Boundary differences fell into three main categories: contig artifacts, intron errors and valid variation. Contig artifacts included ORFs running off the end of contigs and/or ambiguous sequence. In these cases, the pipeline called the closest available in-frame start or stop. Errors due to introns were overwhelmingly in genes that code for ribosomal proteins, especially those in which the leading coding portion is ∼20 nucleotides or less (e.g. *RPL2A*/YFR031C-A and *RPL2B*/YIL018W). The remainder of the boundary differences appear valid upon viewing the sequence; we know that some start and stop codons differ between strains (e.g. *AQY1*/YPR192W as described in Song *et al.*, (15)). As further experimental data become available, we will refine these annotations appropriately.

**Table 1.** Numbers of automated ORF annotations for 11 different *Saccharomyces* strains for which the predicted translation start and/or stop generated by the AGAPE sequence analysis pipeline (9) differed from the S288C reference

			Automated ORFs calls	ORF boundary differences relative to strain S288C			
Strain	Provenance	Accession		Start	Stop	Both	Total
CEN.PK	Lab strain	JRIV00000000	5379	35	19	39	93
D273-10B	Lab strain	JRIY00000000	5383	37	18	40	95
FL100	Lab strain	JRIT00000000	5366	29	21	34	84
JK9-3d	Lab strain	JRIZ00000000	5385	40	11	35	86
RM11-1a	Vineyard	JRIP00000000	5323	36	17	30	83
SEY6210	Lab strain	JRIW00000000	5400	44	23	26	93
Sigma1278b	Lab strain	JRIQ00000000	5358	31	20	28	79
SK1	Lab strain	JRIH00000000	5350	38	22	32	92
W303	Lab strain	JRIU00000000	5397	54	24	33	111
X2180-1A	Lab strain	JRIX00000000	5387	37	24	35	96
Y55	Lab strain	JRIF00000000	5359	39	26	32	97
Total				420	225	364	1009

Step 2: multiple calls and paralogs. We examined ORFs that had been called on more than one contig (Table 2). Through manual review we selected the best call for each ORF in this set based on contig size and sequence quality, then discarded the unneeded duplicate, triplicate or quadruplicate annotations. We also looked at calls for ORFs that are members of paralogous pairs, which accounted for many of the duplicates. The *S. cerevisiae* genome contains over 500 sets of paralogs (11–13). Often the automated pipeline annotated both paralogous ORFs as the same paralog. These annotation errors are easily identifiable based on up- and downstream neighbors. The majority of ORFs identified erroneously during the automated annotation were those coding for ribosomal proteins (e.g. *RPL33A*/YPL143W and *RPL33B*/YOR234C), chaperones (e.g. *SSB1*/YDL229W and *SSB2*/YNL209W) and transporters (e.g. *GEX1*/YCL073C and *GEX2*/YKR106W; *VBA3*/YCL069W and *VBA5*/YKR105C).

**Table 2.** Numbers of ORFs in 11 different *S. cerevisiae* strains that the AGAPE sequence analysis pipeline (9) called on more than one contig

	ORFs called on multiple contigs		
Strain	Two contigs	Three contigs	Four contigs	Total
CEN.PK	15	3	4	22
D273-10B	14	6	3	23
FL100	12	4	3	19
JK9-3d	8	3	3	14
RM11-1a	12	3	3	18
SEY6210	19	3	4	26
Sigma1278b	14	2	3	19
SK1	15	2	5	22
W303	36	1	2	39
X2180-1A	15	2	3	20
Y55	25	2	5	32
Total	185	31	38	254

Step 3: superfluous contigs. Due to the nature of high-throughput sequencing, a number of redundant contigs were generated for each of the 11 genomes. Because they unnecessarily complicate the genome annotation, they have been removed from the sequence files, reducing the number of contigs by more than half (Table 3). These contigs included those on which no genes were called, most often due to short overall length (e.g. JRIP01000320.1 from strain RM11-1A) or ambiguous sequence (e.g., JRIT01000109.1 in strain FL100).

**Table 3.** Numbers of contigs for 11 different *S. cerevisiae* strains in the original automated output from the AGAPE sequence analysis pipeline (9) and in the curated contig set after manual review

	Contig set	
Strain	Original	Curated
CEN.PK	389	189
D273-10B	403	203
FL100	402	174
JK9-3d	431	197
RM11-1a	325	169
SEY6210	366	183
Sigma1278b	451	206
SK1	389	214
W303	415	236
X2180-1A	409	212
Y55	413	198
Total	4393	2181

Step 4: RNAs and chromosomal elements. One limitation of the automated AGAPE annotation is that it focuses solely on protein-coding genes. Many other types of genes and chromosomal elements can be identified through BLAST, then verified and refined through manual curation. We have identified all 16 centromeres in all 11 strains, and are currently working to identify the many RNA genes (e.g. snRNAs, snoRNAs, tRNAs) and replication origins. This curation work will be ongoing over the coming months.

Step 5: the undefined. We are currently working to identify the 1699 ORFs that were marked ‘unidentifiable’ through the AGAPE pipeline (Table 4). Some of these are indeed identifiable but need annotation updates to account for missed introns and coding segments (e.g. *TDA5*/YLR426W). We expect that the majority of the undefined are actually known ORFs that will ultimately be deleted from the annotation due to ambiguous sequence (e.g. *ALD2*/YMR170C) or because they are truncated at the ends of contigs, often in most or all 11 strains (e.g. *SSA1*/YAL005C and *COS7*/YDL248W). At least some of the undefined represent *bona fide* novel ORFs (e.g. YER065W-A in strain JK9-3d).

**Table 4.** Numbers of ORFs 11 different *S. cerevisiae* strains that were marked as ‘unidentifiable’ in the original automated output from the AGAPE sequence analysis pipeline (9). These ORFs are currently undergoing manual review

Strain	Undefined ORFs
CEN.PK	169
D273-10B	254
FL100	128
JK9-3d	121
RM11-1a	344
SEY6210	69
Sigma1278b	106
SK1	124
W303	158
X2180-1A	78
Y55	148
Total	1699

Steps 6 and 7: omissions and supercontigs. Curation work in the coming year will focus on annotating essential, conserved protein-coding genes that we expect are present in the other genomes but escaped automated annotation undetected. We will also assess several contig pairs for the possibility of combining them into supercontigs (e.g. contigs JRIU01000255.1 and JRIU01000122.1 from strain W303).

## Future Directions

The expansion in SGD from a single reference genome to a multi-genome reference panel furthers yeast genomics research by providing easy access to alternative alleles and sequence variants (14). The automated annotations for the additional genomes, as produced by the AGAPE pipeline, have already been incorporated into SGD sequence and alignment pages, analysis tools such as BLAST, PatMatch, and the Variant Viewer, and are also available for download (Table 5). The application of manual curation to automated output improves the quality and increases the depth and granularity of genome annotation. Curated sequence files and annotation will be incorporated into SGD and also submitted to NCBI’s GenBank primary sequence repository within the coming year. As further research by the scientific community provides updated information, we will incorporate improved annotations into future genome releases. Consideration will also be given in the future to the possibility of expanding the reference panel to accommodate emerging or underserved areas of study, as part of our continuing efforts to educate students, enable bench researchers and facilitate scientific discovery.

**Table 5.** Additional *S. cerevisiae* strain genome sequences are already available throughout SGD

Location	URL
Alignment pages	http://www.yeastgenome.org/cgi-bin/FUNGI/alignment.pl?locus=sal1
BLAST	http://www.yeastgenome.org/blast-sgd
Downloads	http://www.yeastgenome.org/download-data/sequence
Sequence pages	http://www.yeastgenome.org/locus/sal1/sequence
Pattern matching	http://www.yeastgenome.org/cgi-bin/PATMATCH/nph-patmatch
Variant viewer	http://www.yeastgenome.org/variant-viewer

## Funding

This work was supported by a U41 grant from the National Human Genome Research Institute at the US National Institutes of Health (HG001315) to the *Saccharomyces* Genome Database project. The content is solely the responsibility of the authors and does not necessarily represent the official views of the National Human Genome Research Institute or the National Institutes of Health.

*Conflict of interest*. None declared.
